# Identification of two novel Chlorotoxin derivatives CA4 and CTX-23 with chemotherapeutic and anti-angiogenic potential

**DOI:** 10.1038/srep19799

**Published:** 2016-02-02

**Authors:** Tengfei Xu, Zheng Fan, Wenxin Li, Barbara Dietel, Yingliang Wu, Matthias W. Beckmann, Jana K. Wrosch, Michael Buchfelder, Ilker Y. Eyupoglu, Zhijian Cao, Nicolai E. Savaskan

**Affiliations:** 1 Translational Neurooncology Lab, Department of Neurosurgery, Universitätsklinikum Erlangen, Friedrich-Alexander University (FAU) of Erlangen - Nürnberg, D-91054 Erlangen, Germany; 2 State Key Laboratory of Virology, College of Life Sciences, Wuhan University, Wuhan, 430072 P.R. China; 3 Translational Research Center, Department of Cardiology and Angiology, Universitätsklinikum Erlangen, Schwabachanlage 12, 91054 Erlangen, Germany; 4 Department of Gynecology and Obstetrics, & Comprehensive Cancer Center Erlangen-EMN, Universitätsklinikum Erlangen, Friedrich-Alexander University (FAU) of Erlangen – Nürnberg, Erlangen, Germany; 5 Department of Psychiatry and Psychotherapy, Universitätsklinikum Erlangen, Friedrich-Alexander University (FAU) of Erlangen – Nürnberg, Erlangen, Germany

## Abstract

Brain tumors are fast proliferating and destructive within the brain microenvironment. Effective chemotherapeutic strategies are currently lacking which combat this deadly disease curatively. The glioma-specific chloride ion channel represents a specific target for therapy. Chlorotoxin (CTX), a peptide derived from scorpion venom, has been shown to be specific and efficacious in blocking glioma Cl^−^ channel activity. Here, we report on two new derivatives (termed CA4 and CTX-23) designed and generated on the basis of the peptide sequence alignments of CTX and BmKCT. The novel peptides CA4 and CTX-23 are both effective in reducing glioma cell proliferation. In addition, CTX, CA4 and CTX-23 impact on cell migration and spheroid migration. These effects are accompanied by diminished cell extensions and increased nuclear sizes. Furthermore, we found that CA4 and CTX-23 are selective with low toxicity against primary neurons and astrocytes. In the *ex vivo* VOGiM, which maintain the entire brain tumor microenvironment, both CTX and CA4 display anti-tumor activity and reduce tumor volume. Hence, CTX and CA4 reveal anti-angiogenic properties with endothelial and angiogenic hotspots disrupting activities. These data report on the identification of two novel CTX derivatives with multiple anti-glioma properties including anti-angiogenesis.

Primary brain tumors derived from glial cells belong to the deadliest forms of cancer in humans. Still, effective anti-glioma therapies with curative outcome are still missing. The lack of powerful chemotherapeutics against malignant gliomas can be attributed to missing targets specific for gliomas sparing neurons and glial cells from toxicity. The heterogeneity of this tumor entity is reflected by its clonal variability and different stem cell sources from which these cells are derived from.

In an attempt to identify Cl^−^ specific blockers, the venom of the scorpion *Leiurus quinquestriatus* revealed a significant, reversible inhibitory activity against reconstituted Cl^−^ channels of the small conductance type^1^,^2^. The scorpion venom blocking activity on single-channel was a first-order binding reaction and revealed the identification of the peptide chlorotoxin (CTX) amongst others as a single Cl^−^ specific peptide blocker^3^. Noteworthy, the scorpion venom is rich of physiologically active substances with polypeptides including neurotoxins and enzymes depicting the main active ingredients. In addition, scorpion venom also includes bio-polysaccharides, hyaluronic acid derivates, serotonin, histamine, histamine-releasing factors and protease inhibitors^4^,^5^. These bioactive polypeptides selectively bind to and modulate specific ion channels of excitable cell membranes. Scorpion-derived peptide toxins can be classified according to their specificity in inhibiting various ion channel receptors. So far, scorpion-derived toxins have been identified which are specifically targeted against Na^+^ channels^6^,^7^, K^+^ channels^8^, Cl^−^ channels^9^ and Ca^2+^ channels^10^. A number of chlorotoxin related peptides have been isolated and identified since 1992^2^.

In a remarkable effort to identify reliable properties selective for gliomas, the Sontheimer group reported on a chloride ion channel activity abundant in malignant gliomas and absent in normal brain tissue^11^,^12^. This glioma-specific chloride channel (GCC) can shape glioma cell morphology, foster proliferation and migration, and regulates apoptosis^13^,^14^,^15^,^16^. The observed findings from above-mentioned studies indicate that inhibition of GCC could be used as chemotherapeutic strategy combating malignant gliomas.

With CTX in hand GCC has first been identified in tumor tissue specimens from patients and has also been detected in glioma cell lines such as U251MG, CH235MG, U373MG, U105MG, D54MG, SK-MG-l and STTGl^13^.

In the present study we searched for potent peptides against cancer cells based on the sequences of scorpion toxins CTX and BmKCT. Thereby, we identified two CTX derivatives, termed CA4 and CTX-23, and investigated the role of these compounds in different established glioma cell lines and its impact on the brain-tumor microenvironment. We show that scorpion toxins decrease tumor-induced neuronal damage and reduce glioma cell growth in a concentration-dependent manner. Both CA4 and CTX-23 peptides inhibit rodent and human glioma cell growth already at low concentrations. Importantly, CTX and its derivatives preserve primary astrocytes and neurons from toxicity. Furthermore, we found that CA4 as well as CTX itself can normalize tumor vessel morphology and vessel density in the peritumoral brain area.

## Methods

### Cell lines

Rat glioma cell line F98 and the human glioma cell line U87 were obtained from ATCC/LGC-2397 (Germany) and were maintained as described before^17^. In brief, cells were cultured under standard conditions containing DMEM medium (Biochrom, Berlin, Germany) supplemented with 10% fetale bovine serum (Biochrom, Berlin, Germany), 1% Penicillin/Streptomycin (Biochrom, Berlin, Germany) and 1% Glutamax (Gibco/Invitrogen, California, USA). Cells were passaged at approx. 80% confluence by adding trypsin after two washing steps with phosphate-buffered saline (PBS) and incubated for 5 min, whereupon they were centrifuged at 900 rpm/for 5 min.

### Scorpion toxins

The peptides CA4 and CTX-23 were designed on the basis of scorpion chloride toxins CTX and BmKCT^18^,^19^. The nucleotide sequences encoding CTX, CA4 or CTX-23 peptides were synthesized, amplified by PCR and then inserted into pGEX-6p-1 plasmid. The recombinant pGEX-6p-1-CTX/CA4/CTX-23 plasmid, confirmed by sequencing, was transformed into *Escherichia coli* Rosetta (DE3) cells and the transformed cells were cultured at 37 °C in LB medium containing ampicillin (100 mg/ml). After reaching a defined cell density which is characterized by an optical density (OD) of 0.6, 1.0 mM isopropyl thio-b-D-galactoside (IPTG) was added to induce expression at 28 °C. Cells were harvested after 4 h and resuspended in 50 mM Tris-HCl and 10 mM Na_2_EDTA (pH 8.0). Supernatant from the bacterial cell lysate was loaded onto a glutathione transferase (GST) binding column. The purified fusion protein was desalted using centrifugal filtration (Millipore) and cleaved by enterokinase (Biowisdom) at 25 °C for 16 h. Protein samples were separated by HPLC on a C18 column (10 × 250 mm, 5 μm) (Elite-HPLC) using a linear gradient from 10% to 80% acetonitrile with 0.1% trifluoroacetic acid for 60 min with detection line at 230 nm. The peptides eluted as a major peak at 21–24% acetonitrile. The molecular mass of the purified peptides was obtained by MALDI-TOF-MS.

### Cell proliferation analysis

Cell proliferation was measured using 3-(4,5-dimethylthiazol-2-yl)-2,5-diphenyl-tetrazolium-bromide (MTT) assay as describe before^20^. Cell death was monitored by propidium iodide (PI) staining. Briefly, cells were seeded at 3000 cells/cm^2^ in 96-well plate and incubated at standard conditions. After 12 h, the cells were treated with scorpion peptides. Forty-eight hours after treatment, cells were incubated with MTT solution (Roth, Karlsruhe, Germany) (5 mg/ml) or PI (Sigma, Germany) (3 μg/ml), at 37 °C, 5% CO_2_. After 4 h MTT incubation or 10 min of PI incubation, images were obtained using with Olympus X71 microscope (Olympus, Hamburg, Germany) with a long-distance 10 × objective. Exposure time was equal in different groups. Images were taken with cell- F software (Olympus). Cells with 4 h MTT incubation were then lysed with 100 μl acidic isopropanol. OD values were determined with a SLT spectra devise (Crailsheim, Germany) using Tecan × Fluor4 software for quantification.

### The VOGiM organotypic glioma invasion model and angiogenesis analysis

Brain slice cultures were conducted from five days old Wistar rat pups. Brains were prepared and maintained as previously described^17^,^21^. Briefly, animals were sacrificed by quick head dissection and brains were removed and kept under ice-cold conditions. Frontal lobe and cerebellum were dissected of the hemispheres. The remaining brain was cut into 350 μm thick horizontal slices using a vibratome (Leica VT 1000S, Bensheim, Germany). Thereupon brain slices were transferred onto culture plate insert membrane dishes with a pore size of 0.4 μm (GreinerBioOne, Frickenhausen, Germany) and subsequently transferred into six-well culture dishes (GreinerBioOne) containing 1.2 ml culture medium (MEM–HBSS, 2:1, 25% normal horse serum, 2% L-glutamine, 2.64 or 14.3 mg/ml glucose, 100 U/ml penicillin, 0.1 mg/ml streptomycin, 10 μg/ml insulin–transferrin–sodium selenite supplement and 0.8 μg/ml vitamin C). The slices were cultured in humidified atmosphere (35 °C, 5% CO_2_). After 24 h, the 6-well plates were washed with 1.2 ml PBS. Thereafter, we added fresh culture medium and started scorpion peptides treatment. At the second day after preparation, 0.1 μl of a tumor cell-medium-suspension was placed onto the cortex of the slice (10,000 F98^GFP^ cells) by using a 1-μl-Hamilton-syringe. The medium was exchanged after tumor implantation and every second day onwards. Propidium iodide (PI) staining was performed at the same time of medium change and slices were incubated with final concentration of 1 μg/ml PI for 15–20 min. Afterwards, slices were washed in 6-well plates with PBS whereupon complete medium was exchanged and microscopic imaging followed. Peptides treatment was performed after complete medium change, 1.2 μl peptides solution were added to the new medium to obtain the final concentration of 5 μM and 10 μM. At day 7 of culture, all slices were fixed in immunofixative solution (containing of 4% formaldehyde and picric acid) and further immunostained for vessel analysis. Tumor size was measured by image J software (NIH, USA). Vessel density quantification was performed by the overlay grid method^22^ as described. In brief, we designed 80 × 80 μm grids to cover the entire vessel images and calculated the number of events in which single vessels crossed the grids.

### Cell morphology analysis

Cells were seeded at a density of 2500/cm^2^ in 12 well-plates on coverslips and cultured under standard conditions. After two days, cells were treated with peptides for another 24 h. Cells were than fixed with 4% PFA and stained with phalloidin (1:200, Molecular Probes, Life Technologies) overnight and Hoechst (1:10000) for additional 5 min. Coverslips were mounted on slides with Immu-Mount (Thermo scientific, Massachusetts, USA). Pictures were taken with Olympus X71 microscope with x1000 magnitude. Exposure time was equal in different cell lines. Images were taken with cell- F software (Olympus, Tokyo, Japan). Cell length and nuclear morphology was measured by image J software (NIH, USA).

### Scratch migration assay

Scratch migration assay was carried out as previously described^23^. In brief, cells were plated in 24 well-plates at 15000 cells/cm^2^ and held under standard conditions until reaching confluency of 80%. Scratch was standardized by using a serotec 200 μl pipette tip. Floating cells were carefully removed by changing medium. The cells were treated with different concentrations of scorpion venoms at the meantime. Images were taken for each scratch immediately after treatment (0 h). Plates were then held under standard conditions for additional 24 h. Pictures were taken on the time points 0 h, 12 h, and 24 h by Olympus IX71 microscope and the data was analyzed with Image J software (NIH, USA) by measuring distance between the migrating cell boundaries.

### Three dimensional spheroid migration assay

For the spheroid assay 100.000 cells were packaged in 100 ul mixture with 32% methylcellulose solution and 68% culture medium. Cell packages were dropped onto the petri dishes and reversely cultured under standard humidified conditions (37 °C, 5% CO_2_) for 12 hs so that the package is stereoscopic with all cells having aggregated to the top and formed spheroid. Afterwards the spheroid was moved to the 48-well plate and kept culturing with medium. After 12 hours, the spheroid attached to the bottom of the well and the cells migrate, from this moment on (0 h), spheroids were treated with different concentration of scorpion venoms. The pictures of spheroids were taking on the time points 0 h, 24 h, and 48 h by Olympus IX71 microscope, and the cell growth area and the distance from the most distant invasion cells to the spheroid center were measured by Image J software.

### HUVEC isolation and tube forming assay

Primary human umbilical vein endothelial cells (HUVEC) were isolated from freshly collected neonatal umbilical cords (Department of Gynecology, University Hospital Erlangen-Nürnberg, Germany). The umbilical veins were flushed with NaCl until blood was completely removed. Afterwards, endothelial cells were enzymatically isolated by 30 min of incubation with a solution containing 2% dispase, which was diluted in PBS. Thereupon, cells were centrifuged and resuspended in endothelial cell growth ECGM medium (Promo Cell), containing 5% fetal calf serum, 4 μL/mL heparin, 10 ng/mL epidermal growth factor, 1 μg/mL hydrocortisone, 50 μg/mL gentamycin sulfate, and 50 ng/mL amphotericin B and cultured in 37 °C, 5% CO_2_ humidified atmosphere at 37 °C. For tube forming assays, 96-well plates were evenly coated with Matrigel (BD Biosciences) on ice, and then placed at 37 °C for 1 hour until the matrigel of each well became solid. Endothelial cells were seeded with 100 μl volume of growth medium for each well. Images were taken with 10 × objective 1 hour after seeding cells. The forming tubes were quantified by angiogenesis plugin of image J software (NIH, USA).

### Primary rodent astrocyte and neuronal cultures

Primary rat astrocytes were isolated from rat whole brains aged P4-P6 without cerebellum (Charles River Laboratories, Wilmington, MA). Brains were freed from meninges and placed in ice-cold HBSS buffer without serum. Then, tissue was further gently triturated with fire-polished Pasteur pipettes of diminishing tip diameter until tissue was uniformly homogenized. Minced brain tissue was trypsinized with 0.25% trypsin for 10 min. After resuspension and centrifugation, astrocytes were resuspended in full DMEM medium (Biochrom, Berlin, Germany) supplemented with 10% fetal calf serum and 100U penicillin/streptomycin (Biochrom). From the following day on astrocyte culture flasks were continuously agitated to separate detaching microglia. For determination of cell purity, astrocytes were plated on glass slides and after 48 h culturing cells were fixed in 4% formalin for 15 min and processed for glial fibrillic acidic protein immunostaining (Dako, Hamburg, Germany). Glial fibrillary acidic protein (GFAP) cells indicated a purity of astrocytes above 90%. Up to passage #7 astrocytes were used for our experiments. For the preparation of hippocampal neuronal cultures we used freshly isolated postnatal Wistar rat brains. Hippocampi were removed from newborn rat brains in ice cold Hank’s salt solution. After trypsin digestion neurons were triturated mechanically and plated in MEM medium, supplemented with 10% fetal calf serum and 2% B27 supplement (all from Life Technologies-Invitrogen, Karlsruhe, Germany). Treatment of neurons was started after 20 days *in vitro* (DIV).

### Primary human GBM cells

Primary human glioma cells were freshly isolated from surgical resections of human GBM specimens. For the establishment of dissociated glioma cells, tissue samples from human patients were dissected by scissors followed by trypsin incubation for 10 min. After centrifugation, cells were resuspended in DMEM/Ham’s F12 with 10% fetal bovine serum, 1% antibiotic-antimycotic mix, (Invitrogen; Karlsruhe, Germany), supplemented with 5 ng/ml bFGF and 5 ng/ml PDGF-AA (PAN-Biotech, Aidenbach, Germany) and plated on dishes. Cells were maintained in a 95% air – 5% CO_2_ humidified atmosphere at 37 °C.

### Ethics Statement

Studies with human tissue were conducted in compliance with the Helsinki Declaration and approved by the Ethics Committee of the Friedrich-Alexander-University of Erlangen-Nuremberg. All patients gave written informed consent to participate in the study. Animal killing was performed in accordance with the German Protection of Animals Act §4 paragraph one and three. The experimental protocols for animal killing were approved by the committee and designee for animal protection of the University of Erlangen-Nuremberg (TS-7/12).

### Statistical significance

Data from experiments were obtained from at least three independent experiments (n ≥ 3) if not otherwise stated. Statistical analysis was performed using MS Excel 2010 (Microsoft Corp., Washington, USA) using the unpaired two-sample t-test if not otherwise specified in the figure legends. The level of significance was set at *P < 0.05 according to the international conventions. Error bars represent ± standard deviation (S.D.).

## Results

### Identification, expression, purification and characterization of two novel CTX derivatives

**Figure 1:**
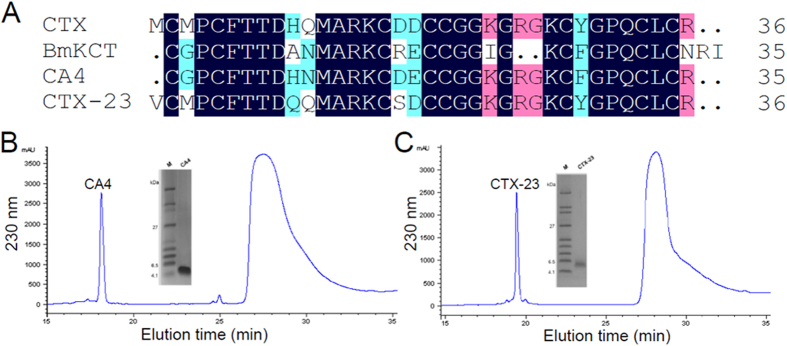
Expression, purification and characterization of two novel CTX derivatives. (**A**) Multiple sequence alignments of Chlorotoxin (CTX), BmKCT, and the two derivatives CA4 and CTX-23. The conserved amino acid residues are highlighted with black background, and the mutated amino acid residues are indicated in color. (**B**) HPLC purification and SDS-PAGE analysis of the derivative peptide CA4. The expressed and purified GST-CA4 fusion protein was digested with enterokinase, after which RP-HPLC was used to separate GST protein and CA4 peptide, corresponding to peaks at elution time from 18 min to 18.5 min and from 27 min to 30 min, respectively. (**C**) HPLC purification and SDS-PAGE analysis of the derivative peptide CTX-23. The expressed and purified GST-CTX fusion protein was digested with enterokinase, after which RP-HPLC was used to separate GST protein and CTX-23 peptide, corresponding to peaks at elution time from 19.5 min to 20 min and from 27 min to 30 min, respectively.

We first generated CTX peptides in the quality suitable for bioassay usage. The recombinant CTX peptide was expressed, purified and characterized according to previous report by Yu *et al.*^24^. Based on the molecular templates of scorpion chloride toxins CTX and Buthus martensii Karsch chlorotoxin-like toxin BmKCT, we designed two novel derivatives to produce the intermediate between CTX and BmKCT (Fig. 1A). The two novel peptides were termed CA4 and CTX-23. The nucleotide sequence encoding CA4 or CTX-23 was generated by overlapping PCR method. The recombinant peptide CA4 and CTX-23 were expressed, purified and identified, using the same method for the recombinant CTX peptide. The GST-CA4 and GST-CTX-23 fusion proteins with a molecular weight of about 30 kDa were expressed and successfully purified and split into two products, the GST gene product portion of the fusion protein at 26 kDa and a small peptide running at 4.0 kDa. The mixture was further separated by HPLC which resulted in two peaks (Fig. 1B,C). The components eluted at about 18 to 20 min corresponding to CA4 or CTX-23 and were collected manually with subsequent lyophilization. The correct molecular weight of CA4 and CTX-23 were further verified by matrix-assisted-laser-desorption/ionization time-of-flight mass spectrometry (MALDI-TOF-MS) (data not shown).

### CTX and its derivatives CA4 and CTX-23 inhibit glioma cell growth in a concentration-dependent manner

**Figure 2:**
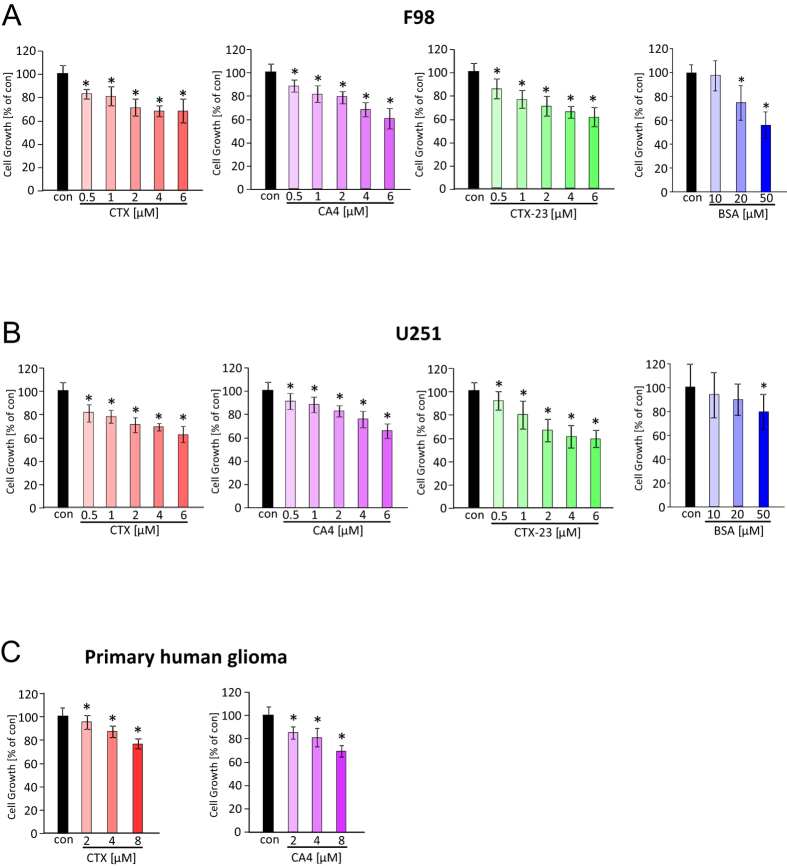
Chlorotoxin (CTX) and its derivatives CA4 and CTX-23 inhibit glioma cell proliferation. Chlorotoxin (CTX) and its derivatives CA4 and CTX-23 were tested on rodent and human glioma cells and on primary human glioma cells clone TN22. (**A**) Cell viability assay of chlorotoxin and its analogues treated rodent gliomas (F98) for 48 hours. BSA served as a control for potential osmotic effects. (**B**) Cell viability assay of CTX and its analogues applied to human gliomas (U251) for 48 hours. BSA served as a control for potential osmotic effects. After the indicated time point, cell viability was determined. (**C**) CTX and CA4 treatment on primary human glioma cells for 48 hours. Quantification is given from three independent experiments. Means are given as ± SD, n = 8 per group, *P < 0.05 (Student’s unpaired t-test).

Next, we analyzed the effects of CTX, CA4 and CTX-23 on glioma cell proliferation. For this purpose we monitored glioma cell growth under various concentrations of scorpion toxin peptides. Here, we found a growth inhibitory effect of CTX, CA4 and CTX-23 already at the lowest concentration of 0.5 μM in rodent and humanglioma cells (Fig. 2). All scorpion venom peptides acted in a concentration-dependent manner and showed a saturation effect at approximately 6 μM (Fig. 2A,B). Noteworthy, CTX-23 and CA4 appeared be most efficacious in inducing glioma cell death compared to CTX (Fig. 2A,B). Bovine serum albumin (BSA) served as a control in these assays. As shown, solely high concentration of BSA above 10 μM showed growth inhibitory effect while 10 μM BSA and lower concentrations were not effective in inducing growth inhibition. Thus, these data imply that a potential osmotic effect caused by the peptides it selves can be excluded (Fig. 2A,B). Further we investigated whether CA4 is also acting on primary glioblastoma cells from human specimens in comparison to CTX. For this purpose we prepared primary glioma cells from a GBM patient and applied CA4 or CTX in the same manner. Interestingly, CTX and CA4 were both effective on freshly isolated primary glioma cells and showed a 30% to 40% reducing effect on cell growth at maximal concentration (Fig. 2C). Furthermore, primary glioma cells appeared to be more sensitive towards CA4 compared to CTX.

### CTX and its derivates CA4 and CTX-23 impact on glioma cell morphology

**Figure 3:**
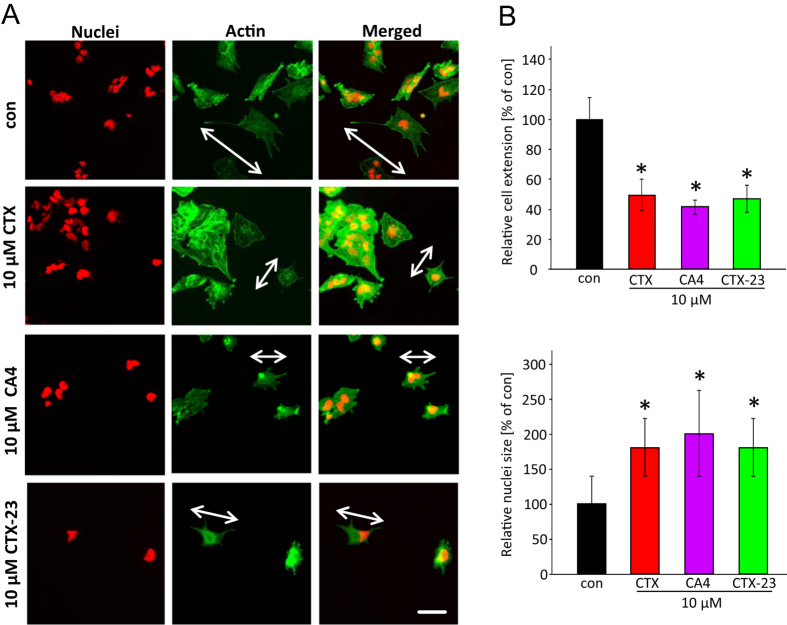
CTX, CA4 and CTX-23 lead to glioma cell shrinkage. (**A**) Human glioma cells U251 were treated with CTX, CA4 or CTX-23 and membrane extensions were monitored. Incubation time for CTX, CA4 or CTX-23 was 24 hours. Nuclei are given in red, actin is displayed in green. Scale bar represents 50 μm. (**B**) Top, Quantification of CTX induced cell shrinkage. Both CTX and its derivatives significantly reduced cell volume compared to controls. Bottom, Quantification of CTX induced nuclei size increase. Both CTX and its derivatives significantly increased the nuclei size of glioma cells compared to controls. Quantification is given from three independent experiments. Means are given as ± SD, n = 10 per group, *P < 0.05 (Student’s unpaired t-test).

In addition, we continued investigating the impact of CTX, CA4 and CTX-23 on glioma cell morphology. Therefore we treated human glioma cells with CTX, CA4 or CTX-23 for 24 hours whereupon cytoskeletal and nuclei changes were monitored (Fig. 3A). After exposure of CTX or CTX-derivatives, the number of cell membrane extensions and filopodia was dramatically decreased (Fig. 3A,B). Noteworthy, this effect was also found to the same extent in CTX treated glioma cells indicating that Cl^−^ channel blockage causes retraction of membrane protrusions. Moreover, the overall cell size atrophied dramatically after treatment. Cell size measurement revealed a 50% to 60% decrease after CTX, CA4 or CTX-23 application (Fig. 3B). Moreover, also the nuclear morphology was also affected (Fig. 3B). After CTX or CTX-derivatives treatment, nucleus of glioma cells increased in diameter and revealed a swollen phenotype (Fig. 3B).

### CTX and its derivatives inhibit glioma cell migration

**Figure 4:**
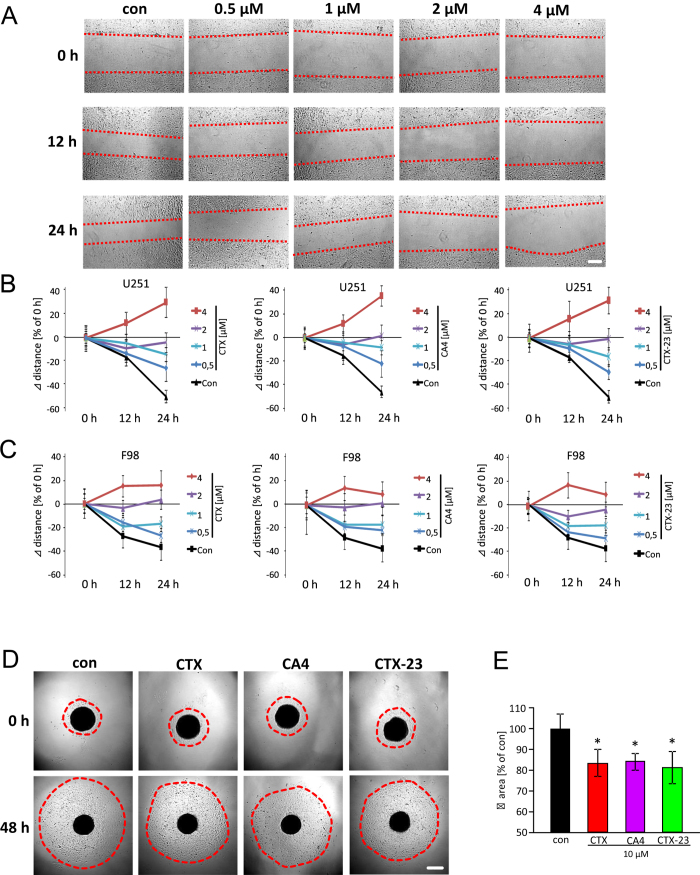
CTX and its derivatives CA4 and CTX-23 inhibit glioma cell migration. (**A**) Representative images of human U251 glioma cell migration was monitored at 0, 12 and 24 hours after scorpion venoms treatment. Low concentration of CTX and its derivatives slowed down the migration and high concentration was able to stop the migration of U251. Scale bar represents 200 μm. (**B**) Quantification of glioma migration after scorpion venom application (CTX, CA4 and CTX-23) at 0 h, 12 h and 24 h. Quantitative values are given as delta distances from the initial distance at 0 h. (**C**) Migration assay of rodent F98 glioma cells. Quantification of glioma migration after scorpion venom application (CTX, CA4 and CTX-23) at 0 h, 12 h and 24 h. Quantitative values are given as delta distances from the initial distance at 0 hr. (**D**) Representative images of three dimensional spheroid migration assay in human U251 glioma cells, cell migration was monitored at 0 and 48 hours after treatment. Scale bar represents 500 μm. (**E**) Quantification of glioma migration area after scorpion venom application (CTX, CA4 and CTX-23) at 0 h and 48h. Quantitative values are given as delta area from the initial spheroid area at 0 hr. Means are given as ± SD, n = 27 per group, *P < 0.05 (Student’s unpaired t-test).

Hence, we tested how CTX and its derivatives affect the motility and migration of glioma cells. For this purpose we performed a migration assay as described previously^23^. Under control conditions, the distance between cell boundaries was reduced to 20% after 12 hours and further reduced to 60% after 24 hours. In stark contrast, CTX and its derivatives reduced cell motility already at a concentration of 0.5 μM (Fig. 4A). Noteworthy, already at 2 μM CA4 and CTX-23 were sufficient to halt glioma motility whereas CTX did not completely suppress migration at this concentration (Fig. 4B,C). At 4 μM all derivatives of CTX completely blocked migration and induced cellular retraction as shown in Fig. 3. Altogether, these results demonstrate that CA4, CTX-23 and CTX act as migratory inhibitors with CA4 revealing the strongest motility inhibitor on various glioma cell species (Fig. 4B,C). This result was further confirmed by three-dimensional spheroid migration assay, 10 μM CTX, CA4 or CTX-23 treatment significantly inhibited the migration of U251 glioma cells (Fig. 4D). Quantification of the invasion area revealed a 20% decrease after venoms treatment (Fig. 4E).

### CTX and its derivatives show no toxic effects to astrocytes and neurons

**Figure 5:**
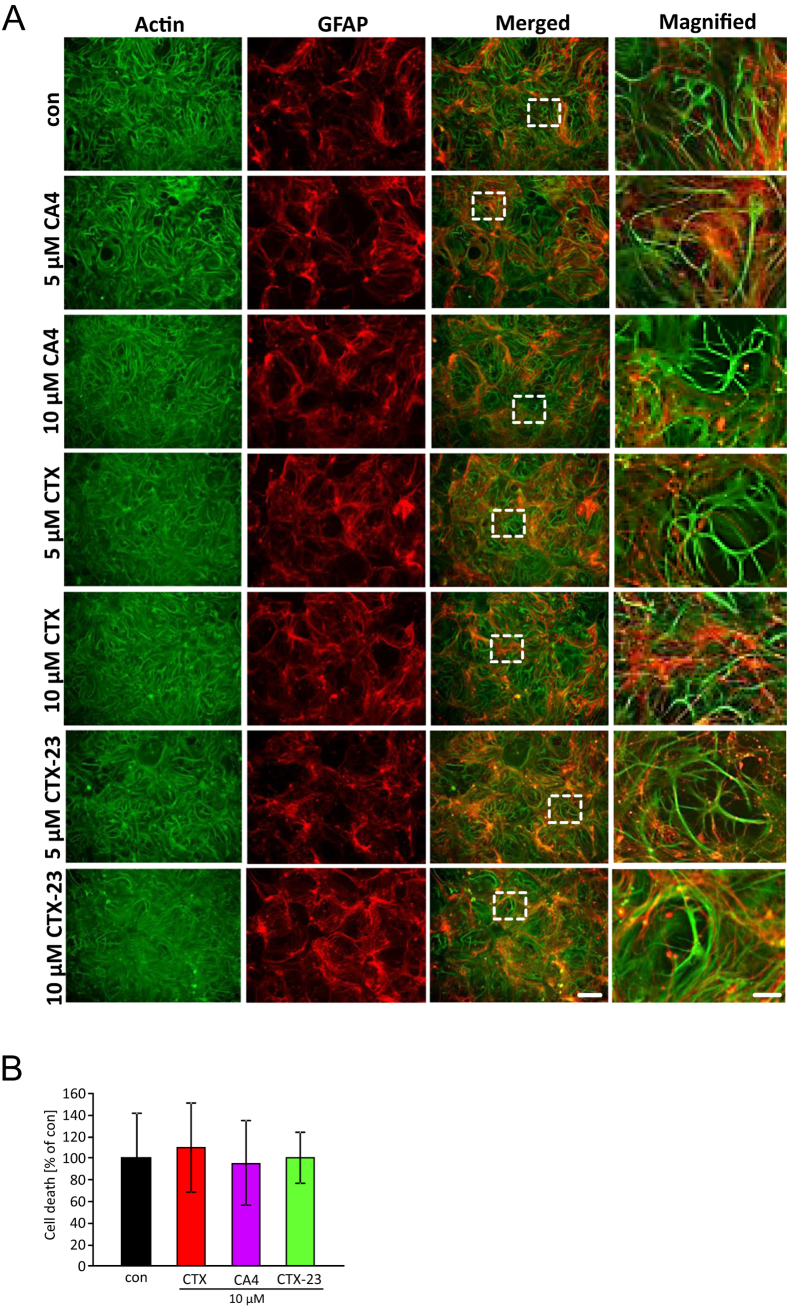
CA4, CTX and CTX-23 toxins do not affect primary astrocytes and neurons. (**A**) Representative images of CTX, CA4 and CTX-23 treated astrocytes and neurons. The duration of treatment was 48 hours. Actin is given in green. GFAP is shown in red. Inserts display higher magnification of merged images. Scale bar represents 100 μm. Scale bar of magnification of inserts displays 20 μm. (**B**) Quantification of PI staining in CTX, CA4 and CTX-23 treated astrocytes and neurons. Means are given as ± SD, n = 5 per group, *P < 0.05 (Student’s unpaired t-test).

We next investigated whether the scorpion toxins CTX CA4 and CTX-23 are toxic in a physiological setting. Therefore we treated primary astrocytes and neurons with these peptides whereupon cell morphology was monitored. In this assays, we applied the toxins in the same manner as glioma cells were treated. Interestingly, CTX and its derivatives showed no toxicity on primary astrocytes and neurons within a wide range of concentration which has been shown to be toxic for glioma cells (Fig. 5A). Also, neuronal and astrocytic cell morphology remained unchanged following chlorotoxin or derivatives treatment with up to 10 μM (Fig. 5A). PI staining revealed that CTX, CA4 and CTX-23 did not induce significant cell death compared to untreated controls (Fig. 5B).

### CTX and CA4 are effective in inducing gliomatoxicity in the VOGiM

**Figure 6:**
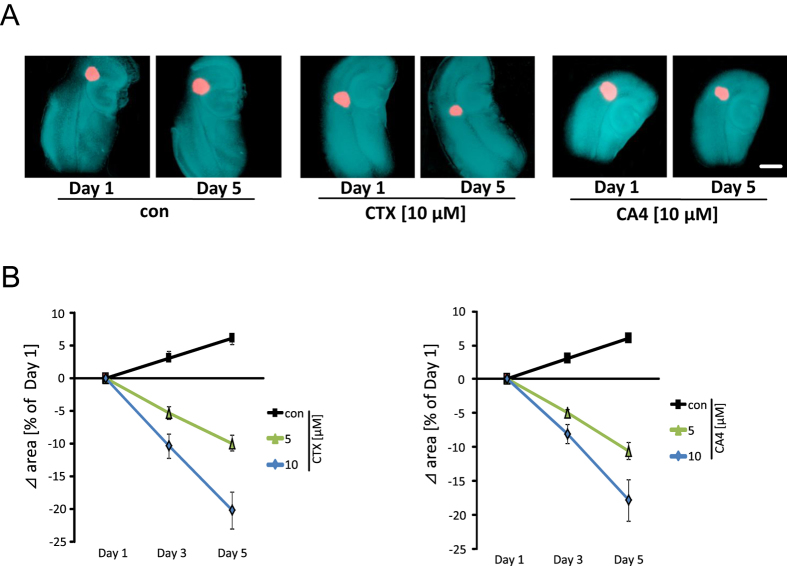
CTX and CA4 toxins execute glioma growth inhibitory effects in brain microenvironment. (**A**) Glioma-implanted brain tissue was treated with CA4 and CTX and tumor spreading was quantified. Treatment started after day 1 to secure initial tumor growth. Brain tissue was treated with CA4 and CTX at 10 μM. (**B**) Quantification of tumor volume in CA4 and CTX treated tumor-implanted brain tissue. Quantification of tumor volume is given. Both CA4 and CTX significantly reduced tumor volume compared to controls. Quantification is given from three independent experiments. Means are given as ± SD, n = 7 per group, *P < 0.05 (Student’s unpaired t-test). Scale bar represents 1 mm.

In order to prove the efficaciousness of CTX and CA4 in an *in vivo* like tumor microenvironment and for comparing their potential differences we facilitated the VOGiM, an *ex vivo* model which enables pharmacological testing in real time mode^22^,^25^. Brain slice sections were cultured on permeable PET membrane bathed in culture medium. One day after glioma cells were implanted into the cortex, brain tissue was treated with CTX and CA4 (Fig. 6). In controls, tumor growth increased within five days and the tumor revealed a demarcated tumor bulk (Fig. 6A). In contrast, glioma-implanted brains treated with CA4 showed a clear tumor regress already at 5 μM (Fig. 6A). CTX treatment was effective to comparable extent as CA4 in inhibiting tumor growth. At 10 μM CA4 and CTX were effective in reducing initial tumor volume by 18 respectively 20% (Fig. 6B).

### CTX, CA4 and CTX-23 operate on endothelial tube forming activity

**Figure 7:**
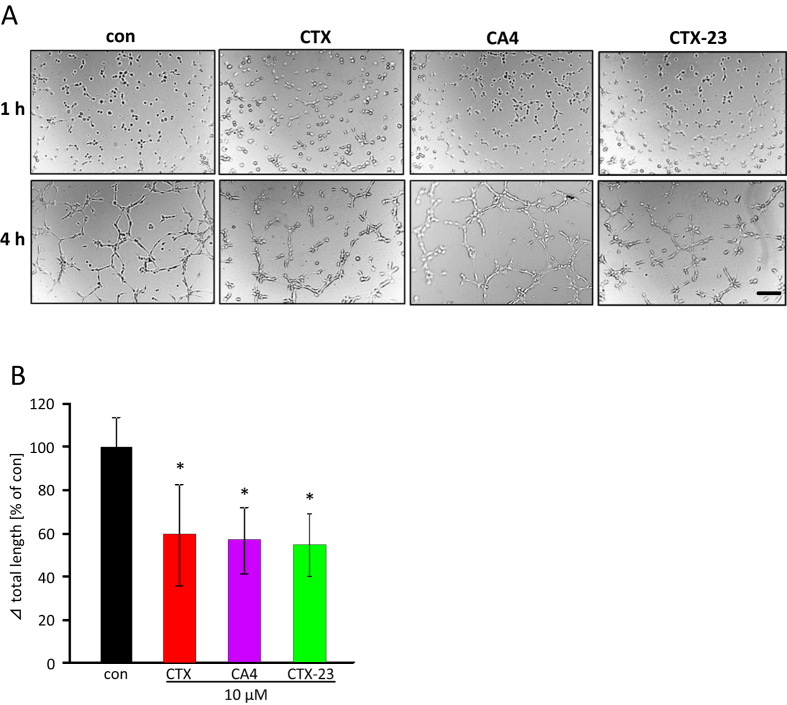
CTX, CA4and CTX-23 toxins reduce tube forming activity in HUVECs. (**A**) HUVEC cells were seeded on matrigel for 1 hr and subsequently treated with 10 μM CTX, CA4 or CTX-23 for another 3 hours. Represented images show tube formation and tube disintegrating activity after toxins application. (**B**) Quantification of the total length of capillary tubes following toxins treatment. Means are given as ± SD, n = 10 per group, *P < 0.05 (Student’s unpaired t-test). Scale bar represents 200 μm.

Since angiogenesis is one major hallmark of gliomas, we examined whether the prototypic glioma-specific Cl^−^ channel blocker CTX and its potent derivatives CA4 and CTX-23 have any effects on endothelial cells. To address this question, we tested these three venoms in endothelial cell tube formation assays. Umbilical vein endothelial cells (HUVEC) were plated on matrigel coated plates whereupon they subsequently received the scorpion venom peptides (Fig. 7). The angiogenic activity was assessed by measuring the length of tubes formed after treatment. Interestingly, 10 μM CTX, CA4 and CTX-23 showed inhibitory effects on tube formation compared to controls (Fig. 7A). The decrease in tube length was 41%, 44% and 46% respectively after CTX, CA4 and CTX-23 application (Fig. 7B).

### CTX and CA4 reduce tumor angiogenesis *ex vivo*

**Figure 8:**
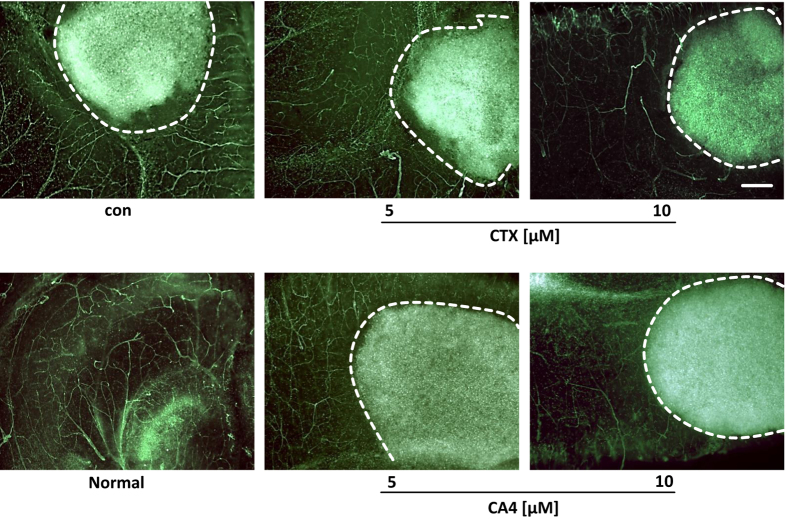
CTX and CA4 inhibit tumor-induced angiogenesis. Tumor-implanted brain slices were treated with CTX or CA4 for 5 days and tumor vessels were monitored. Control brain sections without tumor implantation and tumor-implanted brain sections without treatment served as controls. Scorpion toxin peptide treatment with CTX and its derivate CA4 reduced vessel density in the peritumor area. Scale bar represents 200 μm.

It has previously been demonstrated that in glioma cells are nourished by pre-existing blood vessels, which further proliferate and undergo hyper-angiogenesis with angiogenic hotspots^26^,^27^,^28^,^29^. Furthermore, these tumor-induced vessels are constantly changing in morphology and in function^29^,^30^,^31^. Since we found that CTX and CA4 are effective in blocking endothelial tube formation and since these vascular challenges are presumably important for glioma growth, we wanted to prove this anti-angiogenic activity in a complex brain tumor microenvironment. We studied whether CA4 and CTX are sufficient in normalizing the vasculature. After incubation with the scorpion venom peptides, we stained for the vessel architecture in tumor-implanted brain tissue (Fig. 8). Untreated glioma-implanted brains showed vessels with often irregular and hyper-vascularized angiogenic spots and capillaries (Fig. 8). In contrast, CA4 or CTX treated brain slices revealed reduced numbers of vessels which also appeared less irregular and less dense (Fig. 8). These data indicate that CA4 as well as parental peptide CTX are potent in reducing tumor-induced angiogenesis.

### CTX and its derivatives are heat and serum stable

**Figure 9:**
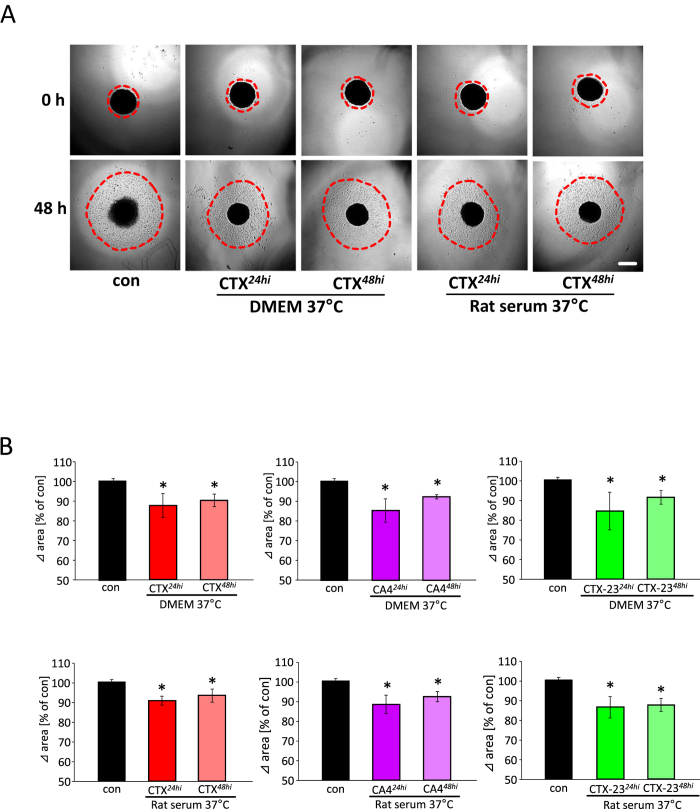
Stability and functional activity of CTX and its derivatives CA4 and CTX-23. (**A**) Representative images of human U251 glioma cell migration monitored at the beginning (0 h)and after 48 hours (48 h) after treatment with pre-incubated CTX peptides in DMEM for 24 hours (CTX^24hi^) and 48 hours (CTX^48hi^) as well as CTX peptides incubated in serum for 24 hours (CTX^24hi^) and 48 hours (CTX^48hi^). (**B**) Quantification of glioma cell migration after medium (DMEM containing 10% serum) or serum pre-incubated scorpion toxin peptide application (CTX, CTX-23 and CA4) at 24 hr and 48 hours. Quantitative values are given as delta distances from the initial distance at 0 hr. Means are given as ± SD, n = XX per group, *P < 0.05 (Student’s unpaired t-test). Scale bar represents 500 μm.

Further, we went on testing the stability of CTX and its derivatives in the presence of the *in vitro* and *ex vivo* conditions. For this purpose we preincubated CTX, CA4 and CTX-23 in culture media based on DMEM containing 10% serum at 37 °C for 24 or 48 hours. In addition we pretreated CTX, CA4 and CTX-23 at 37 °C in 100% rat serum for 24 or 48 hours (Fig. 9). Following the preincubation we tested the functionality of these peptides in the spheroid migration assay as described above. Compared to untreated controls (con), the cell invasion area was reduced by 13% in 24 hrs DMEM pre-incubated CTX peptides and 10% reduced in 48 hrs DMEM pre-incubated CTX (Fig. 9A,B). Next we tested the functionality of rat serum pretreated CTX which revealed that cell invasion area was reduced by 10% in 24 hrs serum pre-incubated CTX and 7% reduced in 48 hrs serum pre-incubated CTX compared with untreated control (Fig. 9A,B). We performed the same experiment with CA4 and CTX-23 (Fig. 9A,B). Thus, the results indicate that CTX, CA4 and CTX-23 were stable and remain functional at least after 24 hrs preincubation in both *in vitro* and *ex vivo* experimental conditions.

## Discussion

Since the identification of Chloride channels (CLC) in various cancer tissue their pharmacological targeting and modulation attracted interests. CLC are majorly divided into voltage-gated and ligand-gated channels which are widely expressed in non-excitable cells, such as erythrocytes, white blood cells, epithelial cells, endothelial cells, and fibroblasts^32^,^33^,^34^. Independent lines of evidence found that malignant gliomas express a specific voltage-gated chloride channel current (GCC) not present in other non-tumor tissues and tumor cells such as rhabdomyosarcoma, melanoma and breast cancer. GCC plays an important role in regulating cell excitability, volume and transmembrane transport, and maintaining cell homeostasis and organelle acidification^35^,^36^. Meanwhile, GCC also plays a role in tumor immune response, proliferation, cell differentiation, invasion and migration^35^. Noteworthy is the finding of chlorotoxin (CTX) as a specific GCC inhibitor^13^,^37^. CTX is a 36 amino acid long peptide derived from the venom of the scorpion *Leiurus quinquestriatus hebraeus* (Deathstalker). We continued to identify CTX analogues in the venom of the scorpion *Mesobuthus martensii* Karsch (Chinese golden scorpion)^18^,^38^ and based on the sequence information designed derivatives.

First, we compared the activity of CA4 and CTX-23 to the established effects of CTX. In our assays we found that CA4 and CTX-23 were equally effective in reducing glioma cell growth as CTX. Also, CA4 and CTX-23 inhibited glioma cell migration and glioma cell morphology. We further tested CA4 in the brain tumor microenvironment and found that CA4 had a high efficacy in inhibiting tumor growth *ex vivo*. Moreover, the novel peptides CA4 and CTX-23 are both not toxic to neurons and astrocytes as is the case for the parental peptide CTX, indicating their specificity towards neoplastic cells. A novel finding is that CA4 as well as CTX both act on endothelial cell function. CA4 reduced the tube forming activity of human endothelial cells. Hence, CA4 and CTX inhibited tumor-induced angiogenesis. Thus, in addition to the aforementioned functions GCC blockers also show a biological effect on endothelial cell function. This is an important fact since CTX has been facilitated in preclinical studies for imaging and targeting gliomas^35^.

Clinically relevant is that CTX can pass the blood-brain barrier^39^,^40^,^41^. Our findings indicate that CTX and its analogues may pass the blood brain barrier (BBB) via direct endothelial cell entry mechanisms. Moreover, malignant gliomas cause alterations in the BBB due to changed vascular permeability leading to a mixed cytotoxic and vasogenic brain edema making targeted drug delivery inefficient^42^,^43^. Therefore, CA4 and CTX-23 can also be function as a delivery enhancer for multimodal chemotherapeutics. So far GCC has not been identified in endothelial cells. Further studies will focus on this important aspect of Cl^−^ channel biology.

Our data revealed that the CTX-based moiety can be widened to two further functional sequences. In addition, CA4 appears to be highly efficacious in inhibiting glioma functions such as growth, membrane extensions and filopodia motility and migration. This information can be crucial in developing CTX-based moieties for designing hybrid molecules with amplified potency. In summary, we report on two novel CTX-based peptide derivatives which are efficient as chemotherapeutics directed against malignant gliomas.

## Additional Information

**How to cite this article**: Xu, T. *et al.* Identification of two novel Chlorotoxin derivatives CA4 and CTX-23 with chemotherapeutic and anti-angiogenic potential. *Sci. Rep.* **6**, 19799; doi: 10.1038/srep19799 (2016).
