# Erratum: Identification of two novel Chlorotoxin derivatives CA4 and CTX-23 with chemotherapeutic and anti-angiogenic potential

**DOI:** 10.1038/srep22206

**Published:** 2016-03-01

**Authors:** Tengfei Xu, Zheng Fan, Wenxin Li, Barbara Dietel, Yingliang Wu, Matthias W. Beckmann, Jana K. Wrosch, Michael Buchfelder, Ilker Y. Eyupoglu, Zhijian Cao, Nicolai E. Savaskan

Scientific Reports 6: Article number: 1979910.1038/srep19799; published online: 02022016; updated: 03012016

The Acknowledgements section in this Article is incomplete.

“We thank Carmen Christoph for excellent technical assistance. This work was supported by the Förderverein des Tumorzentrums Erlangen, funding grants from National Science Fund for Excellent Young Scholars China (No. 31422049) Hubei Science Fund for Excellent Scholars China (No. 2015CFA042) and Sino Kazakh inter governmental scientific and technological cooperation project to Z Cao.”

should read:

“We thank Carmen Christoph for excellent technical assistance. This work was supported by the Förderverein des Tumorzentrums Erlangen, funding grants from National Science Fund for Excellent Young Scholars China (No. 31422049) Hubei Science Fund for Excellent Scholars China (No. 2015CFA042) and Sino Kazakh inter governmental scientific and technological cooperation project to Z. Cao. T. Xu performed the present work under supervision of Dr. Nicolai Savaskan in fulfillment of the requirements for obtaining the degree Dr. med. at the Friedrich-Alexander University of Erlangen-Nürnberg (FAU). We acknowledge gratefully support by the Friedrich-Alexander-University of Erlangen-Nürnberg (FAU) and the German Research Council (DFG) for patronizing the ‘Open Access Publication’ project.”

