# Corrigendum: Identification of two novel Chlorotoxin derivatives CA4 and CTX-23 with chemotherapeutic and anti-angiogenic potential

**DOI:** 10.1038/srep26630

**Published:** 2016-05-25

**Authors:** Tengfei Xu, Zheng Fan, Wenxin Li, Barbara Dietel, Yingliang Wu, Matthias W. Beckmann, Jana K. Wrosch, Michael Buchfelder, Ilker Y. Eyupoglu, Zhijian Cao, Nicolai E. Savaskan

Scientific Reports 6: Article number: 1979910.1038/srep19799; Published online: 02022016; Updated 05252016

This Article contains typographical errors in the legend of Figure 9.

“n = XX per group”

should read:

“n = 3 per group”

